# Mesh suture repair of rectus diastasis with and without a concurrent ventral hernia

**DOI:** 10.1007/s10029-025-03409-5

**Published:** 2025-07-14

**Authors:** Megan M. Perez, Taaha Hassan, Paige N. Hackenberger, Gregory A. Dumanian, Michael Shapiro

**Affiliations:** 1 Division of Plastic Surgery, Northwestern Feinberg School of Medicine, Chicago, IL USA; 2 Department of Surgery, Northwestern Feinberg School of Medicine, Chicago, IL USA

## Abstract

**Purpose:**

The optimal technique for rectus diastasis (RD) repair, particularly when a ventral hernia is present, remains undefined. Mesh suture is a novel device designed to resist suture pull-through in high-tension closures. This study evaluates the technical feasibility and early outcomes of mesh suture use for linea alba plication during open abdominoplasty, with and without concurrent ventral hernia repair.

**Methods:**

A retrospective review of consecutive cases of abdominoplasty with mesh suture linea alba plication was performed between January 2023–2025. Patients who underwent concurrent procedures (hernia repairs, hysterectomies, and tumor excisions) were included. Patients were excluded if planar mesh was used.

**Results:**

Forty-seven patients met inclusion criteria. The average BMI was 26.6 and mean age was 47.9 years. The mean RD width was 5.0 cm. 51% of patients had an existing preoperative ventral hernia and 61.7% of patients underwent a concurrent procedure. Most patients had a standard low transverse incision (68.1%). There was one superficial infection (2.1%), four seromas (8.5%), one hematoma (2.1%), and four soft tissue breakdowns (8.5%). There were no chronic draining sinuses, fistulae, chronic pain, or suture palpability reported. There was one early hernia recurrence (4.2%) among a patient with a preoperative hernia.

**Conclusions:**

Mesh suture appears to be a feasible option for RD plication in patients undergoing abdominoplasty with and without concurrent hernia repair or other abdominal procedures and was associated with low short-term complication rates in this small, single-surgeon series. These early findings, while encouraging, reflect a heterogenous and limited cohort, and follow up was insufficient to assess long-term recurrence, particularly in patients with hernias > 1 cm. As an initial feasibility study, this work supports further investigation in larger, well-controlled cohorts, ideally stratified by hernia size and with prospective, long-term follow up, to evaluate the durability, safety, and generalizability compared to standard practices.

## Introduction

Rectus diastasis (RD) plication for the length of the abdominal wall is a surgical technique during abdominoplasty that has been shown to successfully, and reliably, shape and tighten the abdominal wall contour in women [1]. Studies demonstrate success with various suturing methods and suture type, using both absorbable and non-absorbable suture [2–6]. The European Hernia Society classified RD severity based on inter-rectus distance, yet despite numerous studies, there remains no consensus on the optimal approach for RD repair, particularly when a ventral hernia is present [7, 8]. High-tension closures, especially of combined RD and hernia repair, pose a technical challenge due to the risk of suture pull-through, a failure mechanism in which excess tension and pressure at the suture tissue interface (STI) leads to early or late failure of the midline approximation. Kohler et al. reported increased recurrence rates for suture repairs of small (< 2 cm) hernias when RD was also present, suggesting mesh augmentation may be beneficial in this scenario [9]. Since then, the European Hernia Guidelines now recommend mesh augmentation for even small 1 cm, full-thickness abdominal wall defects; however, these guidelines do not specifically address the context of simultaneous RD repair [8]. 

Duramesh (MSI Chicago, IL), or mesh suture, is a novel suture designed to improve the distribution of forces at the STI, decreasing suture pull-through and enhancing the longevity of repairs [10–12]. Mesh suture is created from 18 strands of fine polypropylene filaments that are braided and bonded; this design allows the suture to flatten orthogonal to the direction of force applied which decreases pressure at the STI and permits fibrovascular incorporation [12, 13]. Mesh suture received its CE Mark in 2021 and approval for marketing by the Food and Drug Administration in 2022. Although mesh suture has been evaluated in select clinical and biomechanical studies, its application for RD repair in the setting of abdominoplasty, particularly with concomitant ventral hernia repair, has not been studied. This study presents early clinical outcomes using mesh suture for RD plication in patients undergoing abdominoplasty, including those with concurrent hernia repair or other abdominal procedures. The objective of this study was to evaluate the feasibility and short-term outcomes of mesh suture in this context and describe the surgical technique used. While limited by a small sample size and short follow up, this initial experience provides early data on technique and complication rates that may inform future, large-scale prospective studies.

## Methods

### Data collection

A retrospective cohort study was conducted and included all consecutive patients with and without hernias who had a full mesh suture RD plication from the xyphoid to the symphysis pubis and abdominoplasty by a single surgeon (G.A.D). The review included cases between January 2023 and January 2025. Patients were identified through institutional implant logs of mesh suture, and all were treated at a single urban, academic hospital. Inclusion criteria included: use of mesh suture for rectus muscle plication in conjunction with abdominoplasty. Exclusion criteria included: use of planar mesh and abdominoplasty without plication. Notably, patients with incisional, epigastric, trocar site, and umbilical hernias were included in the “with hernia” group for analysis. All patients paid a portion of the surgical fee “out of pocket” for the abdominoplasty.

Retrospective review of the electronic medical record (EMR) was performed to evaluate patient characteristics, surgical details, and outcomes. The primary outcome for this report was incidence of surgical site infection (SSI) which includes superficial, deep, and organ space infections. We also collected surgical site events (SSE) including seroma, hematoma, soft tissue breakdown, cellulitis, suture granuloma, chronic draining sinus, and enterocutaneous fistula per definitions by Majumder et al. [14] SSI, SSE, readmissions, and reoperations were recorded within one year of index surgery, however in cases where follow-up extended beyond 365 days, additional review of the medical record was performed to assess for any mesh-related late complications. Hernia formation after index surgery was recorded for longest follow up time available. Mean follow up was calculated from the index procedure to date of last documented review of the electronic medical record (EMR). Hernia recurrence was defined as documentation of a recurrent defect based on physical exam or cross-sectional imaging. The EMR was reviewed for documented follow-ups, including both the internal EMR and “Care-Everywhere,” which allows for a limited view of outside hospital encounters at participating hospitals. As mesh suture was used as part of standard clinical practice by the surgeon, patients did not give additional informed consent.

### Data analysis

Statistical analysis was performed using IBM SPSS Statistics 29 (Armonk, NY: IBM Corp). Descriptive statistics were performed to summarize patient characteristics, surgical details, and outcomes. Univariate subgroup analyses were performed using Chi-square with post-hoc adjusted standardized residuals (cutoff for significance of $\:\pm\:$ 1.96) for categorical variables. For continuous variables, independent sample T-test was performed. A value of *p* < 0.05 was considered statistically significant unless otherwise stated.

### Surgical technique

#### Suture selection and usage

Empirically, the number 2 mesh suture is used for patients with a BMI > 30 or with larger (> 3 cm wide) hernias, as it provides greater tensile strength and better pore stability under higher tension, which may be required in less compliant abdominal walls. The number 1 mesh suture, which consists of finer filaments, and may be less palpable, is preferred for patients with BMI ≤30 or with smaller hernias, where closure tension is expected to be lower and soft tissue coverage is thinner. This selection is based on practical surgical considerations rather than formal comparative testing. Needle size (small or large) is at the preference of the surgeon.

#### Low transverse incision

**Fig. 1:**
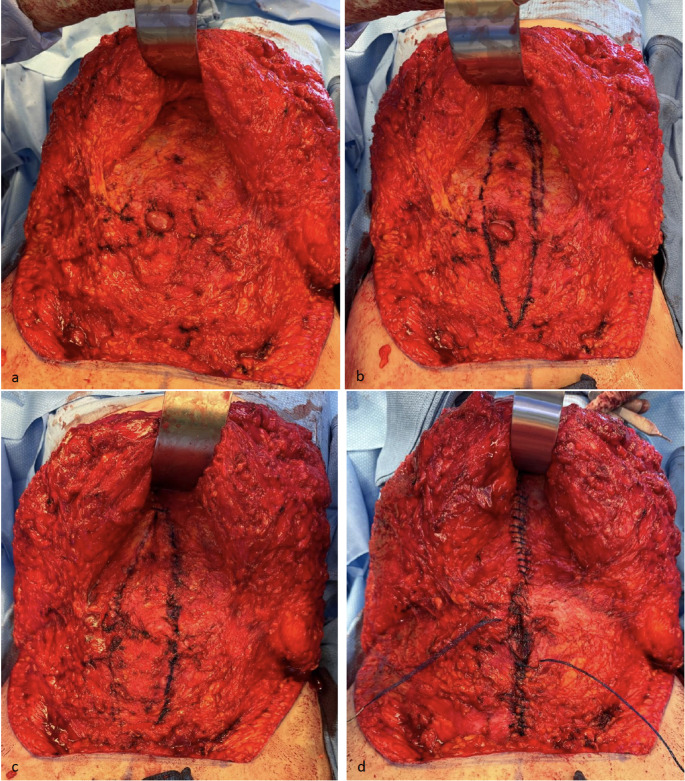
Surgical technique of mesh suture plication of 38-year-old woman with extraction site hernia and severe RD with low transverse incision (**a**) abdominal skin flaps lifted revealing diastasis of the abdominal wall and an incisional hernia (**b**) medial border of anterior rectus fascia is marked with surgical pen (**c**) incisional hernia is closed transversely prior to plication with absorbable, monofilament suture (**d**) plication of linea alba completed with two running mesh sutures that are then tied together and buried at the middle

A long transverse incision is made 7 cm cephalad to the introitus, often through the superior most aspect of pubic hair. Abdominal skin flaps are elevated at the level of Scarpa’s fascia laterally and on the abdominal wall fascia centrally. This is thought to help decrease later seroma formation. The skin flaps are elevated to the level of the umbilicus and then the umbilical stalk is delivered from the tissue with full splitting of the lower skin in the midline. Elevation continues in the midline to expose the linea alba/RD, and then further laterally as needed for anterior rectus fascia plication and later skin redraping. It is not unusual to elevate 10–12 cm of epigastric skin for patients with wide diastases, and there is wider undermining in the infraumbilical than in the supraumbilical region. A midline plication was performed with a running mesh suture (Fig. 1).

The medial border of the anterior rectus fascia is marked with a surgical pen, and then a running closure with 8 mm bites and 8 mm travels between bites is performed to bring the borders of the anterior rectus fascia to the midline and to transpose the stretched-out linea alba posteriorly. Typically, the entry point of the needle is placed 8 mm lateral to the marked medial border of the anterior rectus fascia, and the exit point of the needle is the lateral edge of linea alba. Plications are performed in one layer with this running suture technique. The repair is started at the xiphoid with a buried knot with five alternating throws for the number 1, and 6 throws for the number 2. If only one mesh suture is used, the running suture is continued avoiding the umbilicus and ending at the symphysis pubis where the final knot is also buried. If two mesh sutures are used, the first suture is tied at the xyphoid; a second suture is started at the symphysis pubis and meets the first suture near the umbilicus where a knot is tied and buried.

**Fig. 2:**
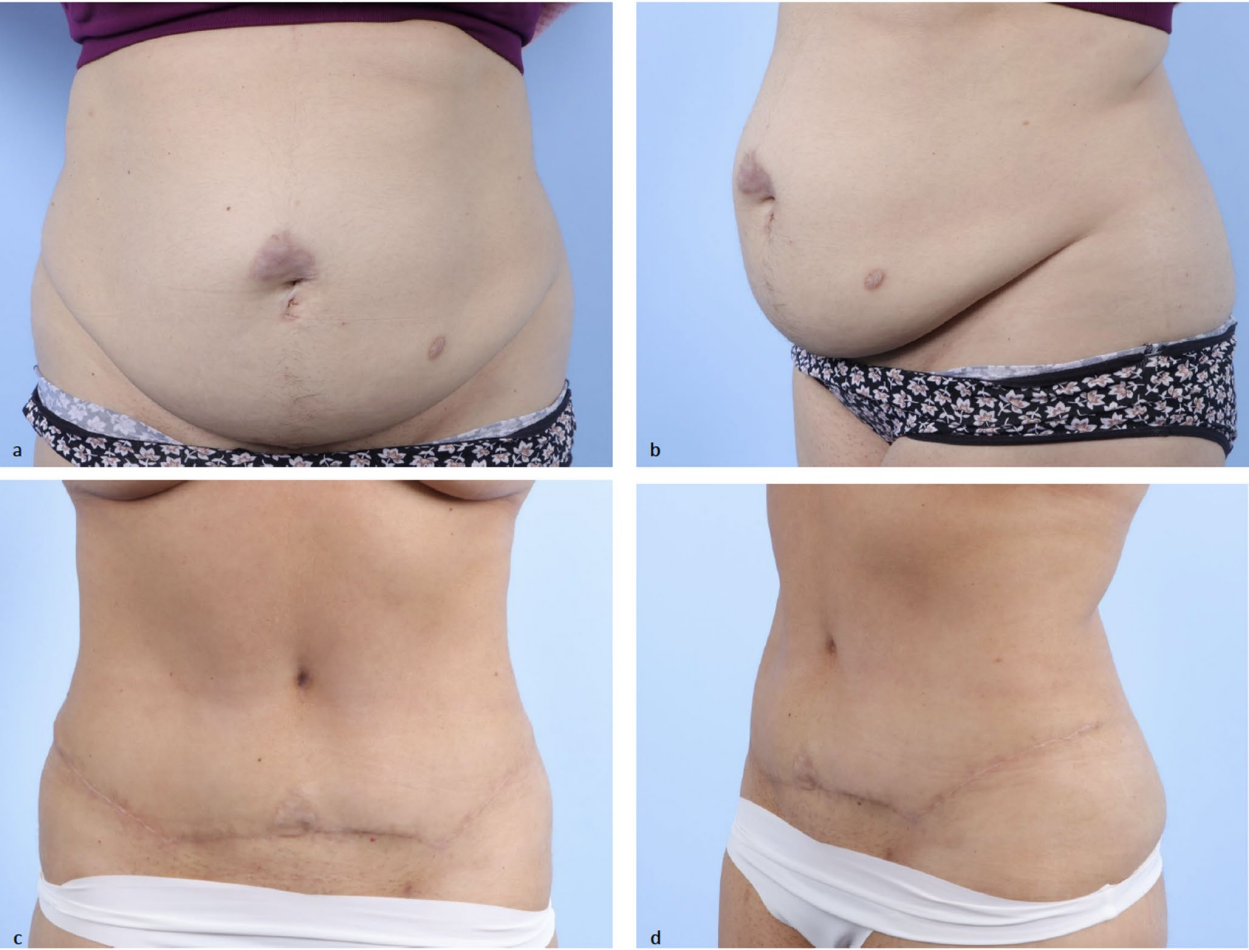
Pre (**a**, **b**) and postoperative (**c**, **d**) clinical photographs of 38-year-old female after abdominoplasty with mesh suture plication (Fig. 1) and extraction site hernia repair with low transverse skin incision

After plication, the patient is placed into 45 degrees of reflux position and skin tailoring is performed. The umbilical stalk is delivered through the abdominal skin flap after first defatting a circular area of 4 cm at the umbilical exit site to create a soft tissue depression in the epidermis. Tissues are irrigated with dilute antibiotic solution. Two drains are placed, and the skin is closed in two layers with 3.0 polyglactin braided suture for the deep dermis and 4.0 monofilament polyglactin suture for the superficial dermis. Drains remain in place until output is less than 30 cc for two days, typically within six to eight days. A single dose of preoperative prophylactic antibiotics is given. Figure 2 demonstrates pre and postoperative photographs of the patient from Fig. 1 who underwent transverse incision abdominoplasty with plication. If the patient has an elevated BMI or a history of deep vein thrombosis, the patient is discharged home on an anticoagulant.

#### Vertical incision

**Fig. 3:**
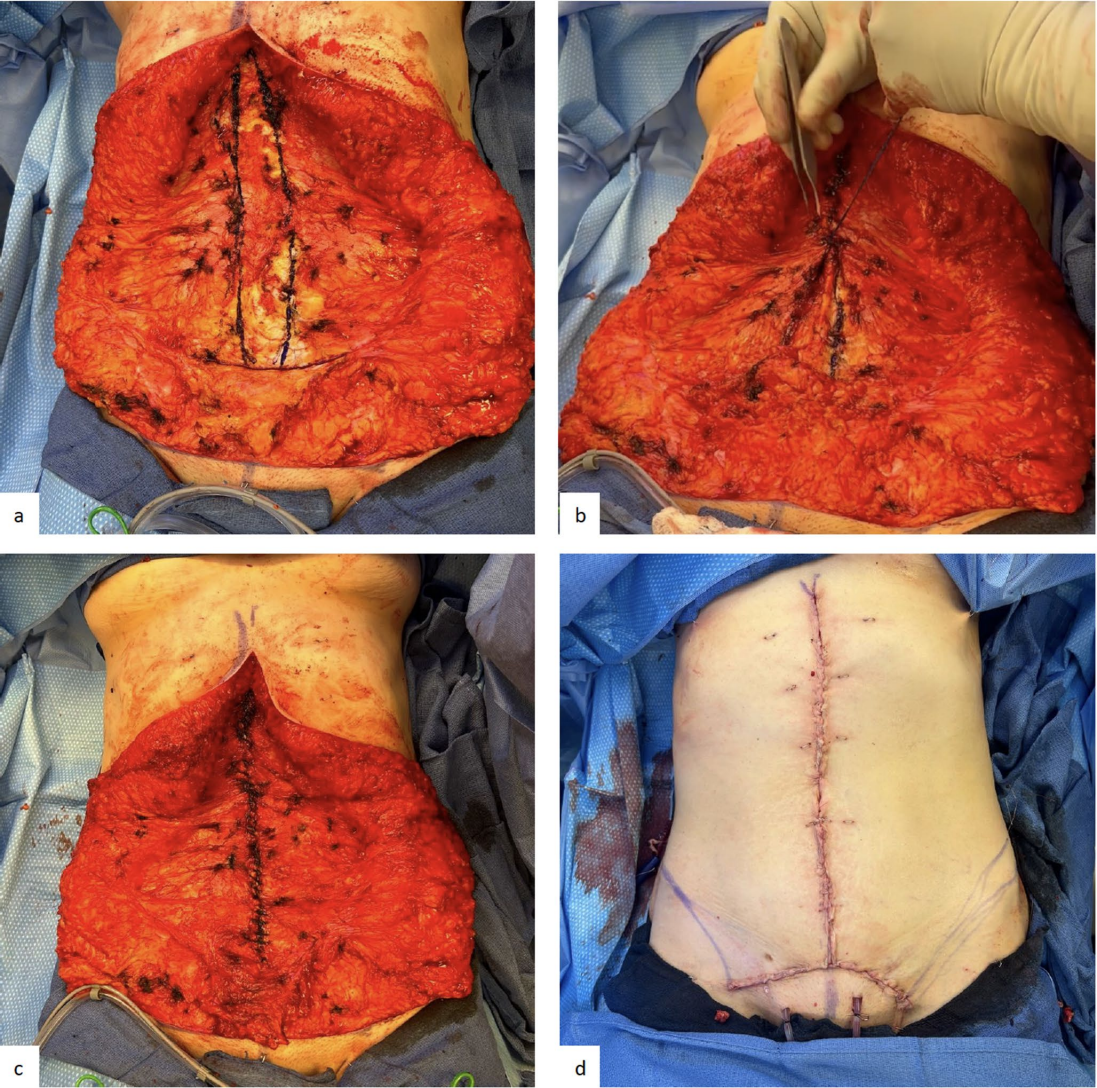
Surgical technique of mesh suture plication of 37-year-old woman with symptomatic umbilical hernia and moderate RD with vertical skin incision (a) abdominal skin flaps lifted revealing diastasis of the abdominal wall and medial border of anterior rectus fascia is marked with surgical pen (b, c) plication of linea alba completed with a single running mesh suture with buried knots (d) skin closure after skin tailoring complete

Transverse marks are placed on the skin to help align the final closure. A wide skin preparation and a wide draping is required, so that at the end closure the surgeon does not assess skin tension to include the weight of the surgical drapes. A long midline longitudinal incision was made from the xiphoid to the pubic area ending 7 cm above the introitus. Abdominal soft tissue flaps are elevated to the semilunar lines bilaterally. The umbilical stalk is excised. The medial borders of the rectus muscle are cleared of soft tissue and a mesh suture is used for plication in a running fashion, in the same manner as described above (Fig. 3).

**Fig. 4:**
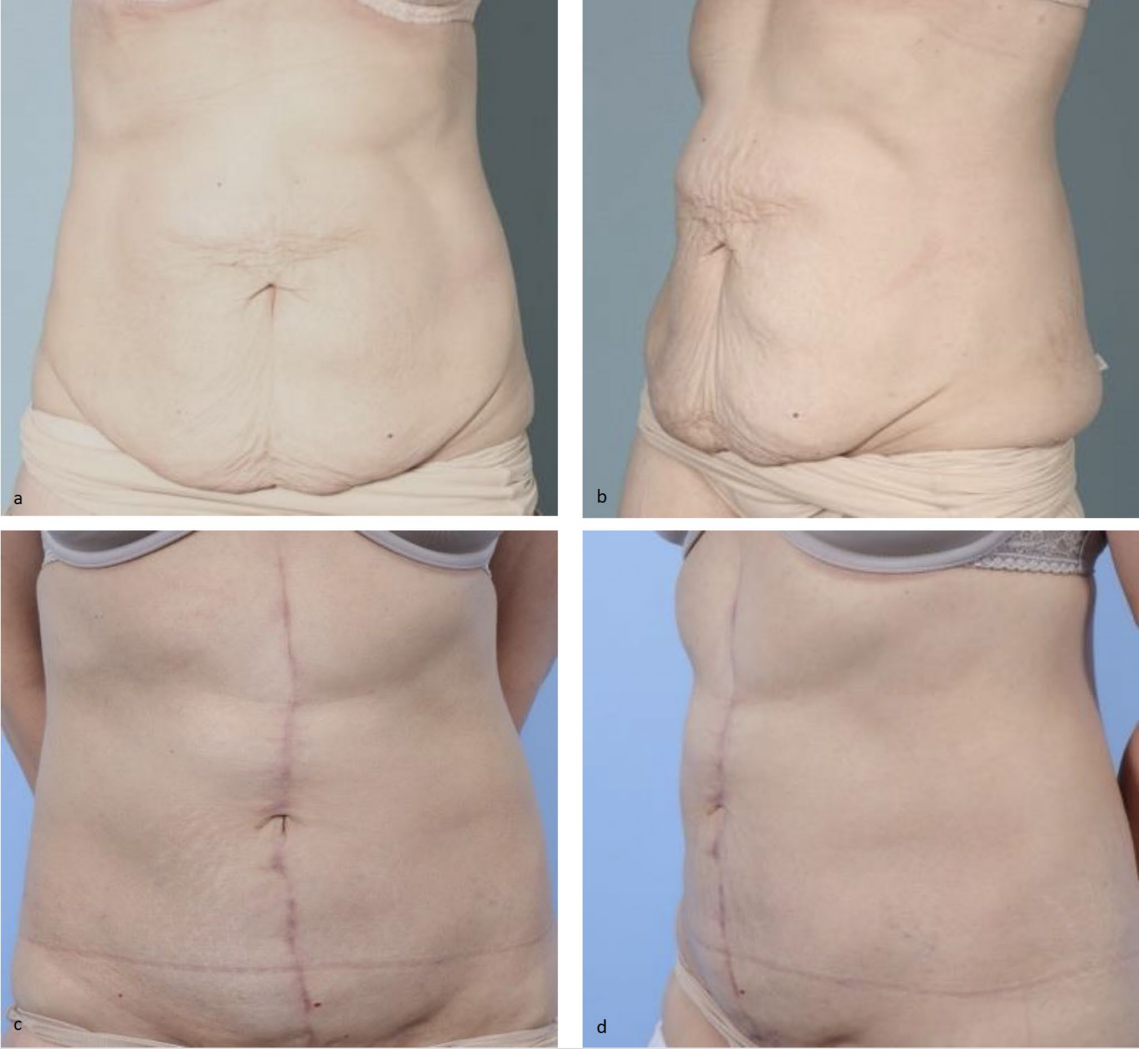
Pre (**a**, **b**) and postoperative (**c**, **d**) clinical photographs of 37-year-old female after abdominoplasty with mesh suture plication (Fig. 3) with vertical skin incision and tailoring

Skin tailoring is performed by pulling the skin flaps downward toward the pubis to decrease the skin dog ear that results superiorly. A neo-umbilicus is fashioned by leaving 2 × 2 cm skin flaps along the skin resection margin. These flaps are tacked down to the abdominal wall at the time of closure [15, 16]. Tissues are irrigated with dilute antibiotic solution. The midline incision is closed with 3.0 polyglactin braided suture for the deep dermis and 4.0 monofilament suture for the superficial dermis. After closing approximately half of the distance from the neo-umbilicus to the symphysis pubis, the patient is sat up 45 degrees and a low transverse incision is made to excise excess skin and soft tissue that results with the lower dog ear. The length of this lower incision depends on specific patient anatomy. Two drains are placed, and the skin is closed in the same fashion as above. Figure 4 demonstrates the pre and postoperative clinical photographs of patient from Fig. 3.

#### Hernia repair

**Fig. 5:**
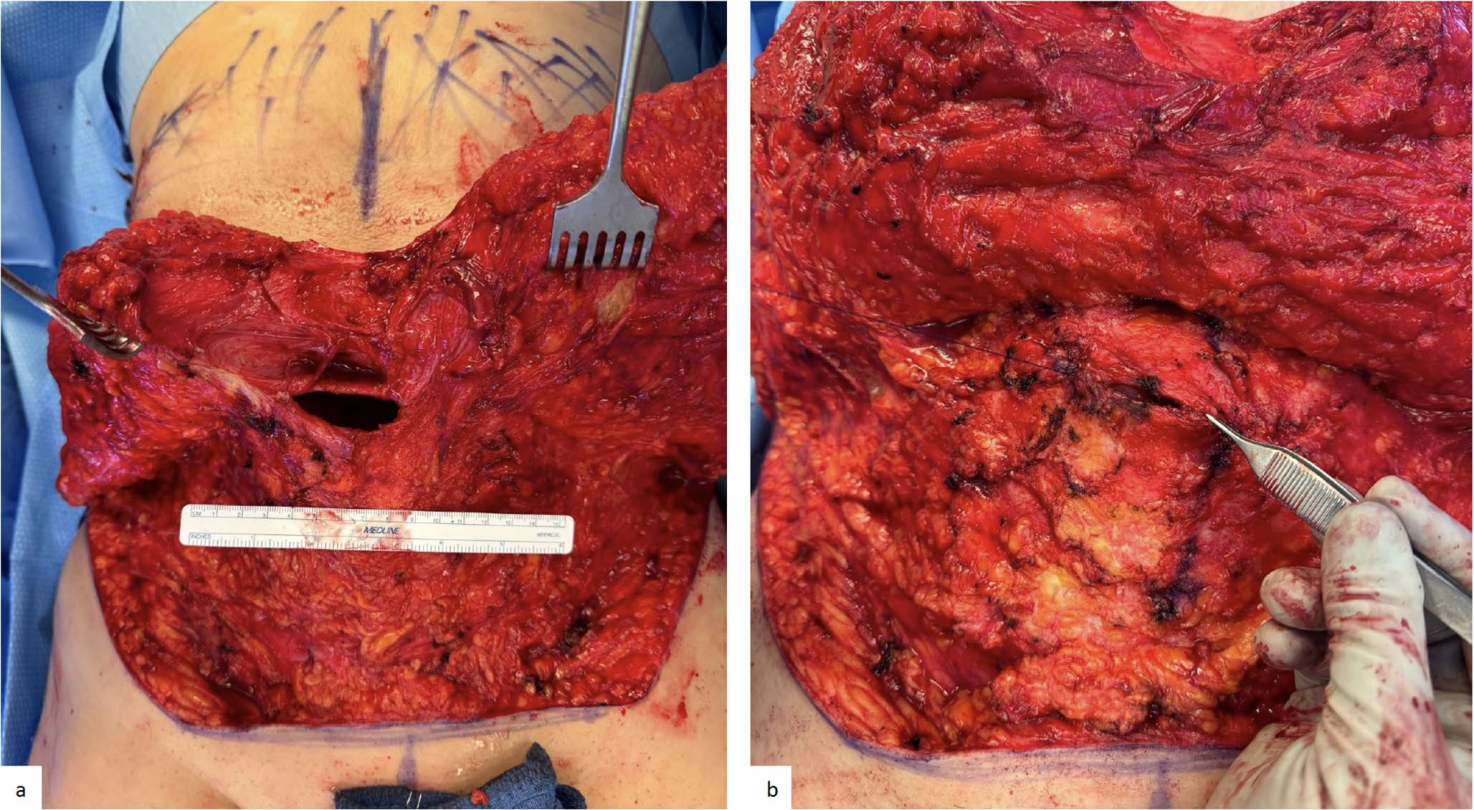
Patient with approximately 4 cm wide extraction site hernia (**a**) soft tissue dissection surrounding hernia defect (**b**) closure of small defect with slowly absorbable monofilament suture, after closure of this defect mesh suture plication of the anterior rectus fascia will occur over defect repair

In cases of epigastric, umbilical, or incisional hernias, the surgeon closes the abdominal wall defect transversely with a slowly absorbable monofilament suture (Fig. 5). Then, the anterior plication is performed as described previously.

For larger incisional hernias, the plication both above and below the defect is the same. However, at the defect itself, there is a transition from an anterior rectus fascial bite to passing the mesh suture full thickness to encompass anterior and posterior fascial layers. In these cases, inclusion of some muscle fibers is intentional, as the well-vascularized muscle may aid in fibrovascular incorporation of the mesh suture, however care is taken to avoid excessive capture or devascularization. The bite size is increased to 10 mm, though 8 mm is still maintained as the spacing between bites. Preperitoneal fat (but not specifically peritoneum) is excluded when possible.

#### Additional skin handling

Depending on skin thickness, liposuction of the epigastrium and the “love handle” area is performed during the procedure with injection of dilute epinephrine/lidocaine solution and suction-assisted lipectomy. Typically, the dilute epinephrine/lidocaine solution is injected at the beginning of the surgery with entry ports for the tumescence cannula designed to minimize risk of intraabdominal puncture. Approximately 500 cc of tumescent solution would be instilled in the epigastrium and 500 cc in the lateral flanks. The liposuction itself is performed after skin flap elevation, again to minimize risk of an intraabdominal puncture.

## Results

Forty-seven consecutive cases of abdominoplasties with mesh suture RD plication were performed between January 2023 and January 2025. Patients had a mean age of 47.9 (± 11.1) years and all patients were female. The mean BMI was 26.6 (± 5.0). The mean RD distance was 5.0 cm (± 2.1). Most patients had at least one prior abdominal surgery (61.7%) and twenty-four patients had a preoperative hernia present at time of surgery (51.1%). The mean width of hernias repaired (when present) was 2.8 cm. Mean length of follow up was 354 days. All patient demographics and surgical details are reported in Table 1, comparing those who underwent concurrent hernia repair to those without using chi-squared and t-tests as appropriate. The only significant differences between patients with and without concurrent hernias were diagnosis of hypertension (*p* = 0.018), BMI (*p* = 0.037) and incision type used (*p* = 0.037).

**Table 1:** Demographics and surgical details of patients undergoing abdominoplasty with and without concurrent hernia repair

	Without Hernia *N* = 23 (48.9)	With Hernia *N* = 24 (51.1)	All patients *N* = 47 (%)	*p*-value
**Age** (mean, SD)	45.2±10.2	50.4±11.6	47.9 ± 11.1	0.112
**Race**				0.094
White	14 (60.9)	19 (79.2)	33 (70.2)	
Black or African American	5 (21.7)	5 (20.8)	10 (21.3)	
Other/prefer not to answer	4 (17.4)	0 (0)	4 (8.5)	
**Gender**				
Female	23 (100)	24 (100)	47 (100)	
**BMI** (mean, SD)	25.1±4.9	28.1±4.8	26.6 ± 5.01	**0.037**
**Current smoker**	0 (0)	1 (4.2)	1 (2.1)	0.322
**Former smoker**	4 (17.4)	6 (26.1)	10 (21.7)	0.475
**Cancer**	5 (21.7)	3 (12.5)	8 (17.0)	0.400
**COPD**	1 (4.3)	0 (0)	1 (2.1)	0.302
**HTN**	4 (17.4)	12 (50.0)	16 (34.0)	**0.018**
**DM**	2 (8.7)	2 (8.3)	4 (8.5)	0.965
**ASA classification**				0.343
I	3 (13.0)	1 (4.2)	4 (8.5)	
II	14 (60.9)	19 (79.2)	33 (70.2)	
III	6 (26.1)	4 (16.7)	10 (21.3)	
**CDC wound classification**				0.322
Clean	23 (100)	23 (95.8)	46 (97.9)	
Clean contaminated	0 (0)	1 (4.2)	1 (2.1)	
**Rectus diastasis width (cm)**	4.9±2.3	5.1±1.9	5.0 ± 2.1	0.856
**Prior abdominal surgeries** (mean, SD)	1.2±0.92	1.3±1.1	1.1±1.0	0.419
**Length of follow up (days)** (mean, SD)	399.1 ± 237.5	311.3 ± 206.2	354.3 ± 224.0	0.182
**Length of inpatient stay (days)** (mean, SD)	0.83±1.0	1.4±1.7	1.1±1.0	0.160
**Hernia History**				
**Prior hernia repair**				
1		7 (14.9)		
> 1		2 (4.3)		
**Pre-operative transverse width (cm)** (mean, SD)		2.8 ± 2.4		
**Pre-operative hernia length (cm)** (mean, SD)		3.5 ± 4.1		
**Surgical details**				
**Operative time (minutes)**	205.4 ± 52.1	182.5 ± 46.4	193.7 ± 50.1	0.119
**Incision type**				**0.037**
Low Transverse	19 (82.6)	13 (54.2)	32 (68.1)	
Vertical	4 (17.4)	11 (45.8)	15 (31.9)	
**Duramesh item used**				
1 SMALL			28 (59.6)	
1 LARGE			10 (21.3)	
2 SMALL			4 (8.5)	
2 LARGE			13 (27.7)	
**Quantity of Duramesh used**				
1			24 (51.1)	
2			20 (42.6)	
3			3 (6.4)	
**Skin closure method**				0.302
Sutures	22 (95.7)	24 (100)	46 (97.9)	
Staples	1 (4.3)	0 (0)	1 (2.1)	

Twenty-nine (61.7%) patients underwent a concurrent procedure (excluding liposuction) at the time of abdominoplasty, which includes hernia repairs. The most common concurrent procedure was umbilical hernia repair (17.0%) and incisional hernia repair (17.0%), followed by epigastric hernia repair (14.9%). Four (8.5%) patients had an abdominal wall endometrioma excised at the time of the procedure and two (4.3%) patients had concurrent open hysterectomies. One (2.1%) patient had a pelvic mass excision. All cases were classified as clean except for one patient with concurrent hysterectomy. Thirty-two patients had a low transverse incision technique used (68.1%) and the remaining 15 patients had vertical incisions (31.9%). Total operative time averaged 193.7 (± 50.1) minutes which included the time for concomitant procedures and abdominoplasty. All cases used a number 1 or number 2 mesh suture. The average length of inpatient stay was 1.1 days.

The outcomes are summarized in Table 2. Overall, there was one (2.1%) superficial infection which accounted for the only SSI reported. Infection was documented as erythema surrounding a drain site that was treated with oral antibiotics and resolved. There were four (8.5%) seromas and one (2.1%) hematoma. All seromas were aspirated in clinic and resolved without reoperation. The hematoma was asymptomatic and managed conservatively. There were four (8.5%) soft tissue incisional breakdowns that were judged to be minor and were managed with local wound care alone. The overall SSE rate was 14.9% at 90 days. Abdominoplasty without concurrent hernia repair as compared to abdominoplasty with concurrent hernia had no significant differences in SSI or SSE outcomes (Table 2). There were no documented patient reports of persistent pain or palpability associated with mesh suture in review of clinical documentation in the EMR.

**Table 2:** Outcomes following mesh suture plication during abdominoplasty with and without preoperative hernia

	Without Hernia	With Hernia	All patients	*p*-value
*N* = (%)	*N* = 23 (48.9)	*N* = 24 (51.1)	*N* = 47 (100)	
**SSI / SSE**				
**SSI**				
Superficial infection	0 (0)	1 (4.2)	1 (2.1)	0.322
Deep infection	0 (0)	0 (0)	0 (0)	
Organ space infection	0 (0)	0 (0)	0 (0)	
**SSI 0–90 days**	0 (0)	1 (4.2)	1 (2.1)	0.322
**Total SSI within 1 year**	0 (0)	1 (4.2)	1 (2.1)	0.322
**Other Complications**				
Seroma	2 (8.7)	2 (8.3)	4 (8.5)	0.965
Hematoma	0 (0)	1 (4.2)	1 (2.1)	0.322
Soft tissue breakdown	2 (8.7)	2 (8.3)	4 (8.5)	0.965
Fascial dehiscence	0 (0)	0 (0)	0 (0)	
Cellulitis	0 (0)	0 (0)	0 (0)	
Suture granuloma	0 (0)	0 (0)	0 (0)	
Chronic draining sinus	0 (0)	0 (0)	0 (0)	
Enterocutaneous fistula	0 (0)	0 (0)	0 (0)	
**SSE 0–90 days**	3 (13.0)	4 (16.7)	7 (14.9)	0.727
**Total SSE within 1 year**	4 (17.4)	4 (16.7)	8 (17.0)	0.947
**Readmission and Reoperations**				
**Readmissions 0–90 days**	0 (0)	1 (4.2)	1 (2.1)	0.322
**Readmissions total within 1 year**	0 (0)	2 (8.3)	2 (4.3)	0.157
**Reoperations 0–90 days**	0 (0)	0 (0)	0 (0)	
**Reoperations within 1 year**	0 (0)	1 (4.2)	1 (2.1)	0.322
**Hernia**				
**Hernia occurrence**	0 (0)	1 (4.2)	1 (2.1)	0.322

There was a single hernia recurrence (4.2%) in a patient who underwent concurrent midline incisional hernia repair and abdominoplasty. Patient had a previous robotic repair of an incisional hernia with an underlay of biologic mesh and had a symptomatic recurrence. During her index procedure she had the previous biologic mesh excised and the hernia was 6.0 cm in length and 5.0 cm in transverse width. The patient’s diastasis and hernia defect were repaired according to the surgical technique described previously. During routine follow up 165 days after index procedure, patient identified an area of softness of the abdominal wall, this was confirmed as a hernia recurrence with CT scan. The patient underwent elective repair of the recurrence which measured 4.0 cm in transversely and 3.0 cm in height. The defect was reclosed transversely with a slowly absorbable monofilament and a RD plication with a running mesh suture that was passed full thickness through the anterior and posterior fascia at the site of the defect, as per our standard technique outlined previously, at 414 days after the index procedure.

There were two patients with readmissions within 365 days of the index procedure and both were unrelated to abdominoplasty. The first patient was readmitted for vaginal bleeding after total abdominal hysterectomy and abdominoplasty. The vaginal cuff was examined under anesthesia with no further bleeding and patient was discharged after one night of observation. The second patient had multiple readmissions for breast cellulitis after breast surgery, unrelated to the abdominal surgery. Regarding reoperations, one patient had a reoperation within 365 days for posterior trunk body contouring with liposuction and revision of the abdominal scar and umbilicus.

Complications were pooled to assess for association with patient demographics or surgical details. Analysis showed no association of any complication with ASA class, smoking status, concurrent procedure, incision type, presence of preoperative hernia, diabetes, hypertension, or cancer history.

## Discussion

This feasibility study describes short-term outcomes of 47 consecutive abdominoplasties with mesh suture RD repair, including patients with and without concurrent ventral hernia repairs. In this heterogeneous cohort, mesh suture repair was associated with low early complications rates. Mesh suture may offer a viable alternative for reinforcing the linea alba in select patients; however, these findings should be interpreted as preliminary given this was a single-surgeon, retrospective case series with short follow-up insufficient to assess hernia recurrence or long-term durability. As such, these results primarily support the technical feasibility and early outcomes profile of mesh suture in this context and provide the foundation for future, larger-scale, prospective investigations.

### Suture repair of RD

It is well known that standard suture has limitations in abdominal wall approximation. Burger demonstrated that for incisional hernias, a suture closure has higher failure rates than a mesh closure with a 60% failure rate at 10 years [17]. In laparotomy closure, even with the small bites technique, failures of tissue approximation occur and increase over the years [18]. Mesh suture plication of the midline is a unique technique distinct from the “small-bites” laparotomy closures that are performed to decrease hernia formation [19]. While the 4:1 concept is well accepted for a standard suture, each mesh suture has 18 filaments that cross the repair site, and it therefore can be considered an exaggerated small-bites technique despite the empirically established 8 mm travels between each suture passage. The capture of the anterior rectus fascia in each bite is also distinct from the passage of the suture only through the linea alba in “small-bites.” The needle of mesh suture has a larger diameter than the 2−0 suture of a slowly absorbable suture and this larger needle size has not been problematic to date in a large registry report of mesh suture [10]. Linea alba plication theoretically should not be different from full thickness abdominal wall closures, other than that the starting point is a compliant female abdomen typically that may be subjected to decreased tissue stresses. However, to achieve an aesthetic outcome, the surgeon may intentionally increase the tension on the repair to better narrow the waistline. Surgeons have described multiple suturing techniques to plicate the linea alba with durable results; many absorbable and nonabsorbable techniques for RD plication are reported in the literature including interrupted and continuous techniques, and multiple layered suture closures [3, 4, 20–26]. While these techniques may be effective in decreasing the tension at the STI, they do require extra time and complexity. Mesh suture may be an alternative option to decrease pressure at the STI while keeping the running plication simple and efficient. Fibrovascular incorporation of the permanent filaments may also lead to a more lasting durable repair.

Marangi et al. was the first to describe the use of mesh suture for RD plication in abdominoplasties, though the study excluded patients undergoing concomitant procedures and hernia repairs [27]. They compared mesh suture to standard 0-polypropylene suture plication and found no significant differences in complications between groups [27]. As compared to our study, we found similar rates of complications including infection (2.1% versus 0%), hematoma (2.1% versus 3.1%), and soft tissue breakdown (8.5% versus 6.2%). Notably we did have more seromas (8.5% versus 1.5%), however half of these were in the concurrent hernia repair group and are attributed to greater soft tissue dissection. It is reassuring that there were no chronic draining sinuses or fistulae in either study. A notable finding by Marangi et al. was that the mesh suture group had a faster plication time as compared to standard suture. This was in part attributed to different suturing techniques used; the mesh suture was used in a running fashion and 0-polypropylene suture was used as interrupted figure-of-eights [27]. The technique used for the 0-polypropylene was likely used due to its propensity for suture pull-through.

### RD repair with hernia repair

Our study is the first to examine the use of mesh suture for abdominoplasty with RD repair and concurrent hernia repair. We observed no significant differences in short-term complication rates between patients undergoing abdominoplasty alone and those undergoing simultaneous hernia repair, suggesting that mesh suture may be a feasible option in both settings. Prior studies have examined the risks and benefits of combining procedures during abdominoplasty, though many are limited by small sample sizes [4, 28–30]. For example, Erfan et al. reported outcomes in 15 patients who underwent anterior component separation and midline hernia repair with absorbable suture and noted a 26.4% complication rate with no hernia recurrences [31]. Moreno-Egea et al. studied 111 patients undergoing concurrent incisional hernia repair with abdominoplasty and found no increase in morbidity compared to either procedure alone, with improvements in quality of life scores [32]. These findings highlight the potential value of integrating surgical approaches, though data remains limited.

Currently, the European Hernia Society recommends mesh reinforcement for umbilical or epigastric hernias > 1 cm in the setting of RD; however, this recommendation is based on low-quality evidence and carries only a weak strength of recommendation [8]. We diverged from these guidelines to explore the use of mesh suture in a patient cohort that may benefit from avoiding planar mesh due to its associated complexity, increased foreign body burden, or potential impact on aesthetic outcomes. Mesh suture, by combining elements of mesh reinforcement with the simplicity of suture placement, may represent a viable alternative for select low-risk patients undergoing aesthetic abdominoplasty with rectus diastasis repair. However, given the small sample size and heterogeneity of our series, particularly in hernia size and type, these findings should be interpreted as hypothesis-generating. Future studies should consider stratified, prospective enrollment, ideally randomized, based on hernia defect size (e.g. <1 cm, 1–2 cm, > 2 cm). Larger hernias may still warrant planar mesh, and repair selection should be guided by individual anatomy, risk profile, and surgical goals.

**Fig. 6:**
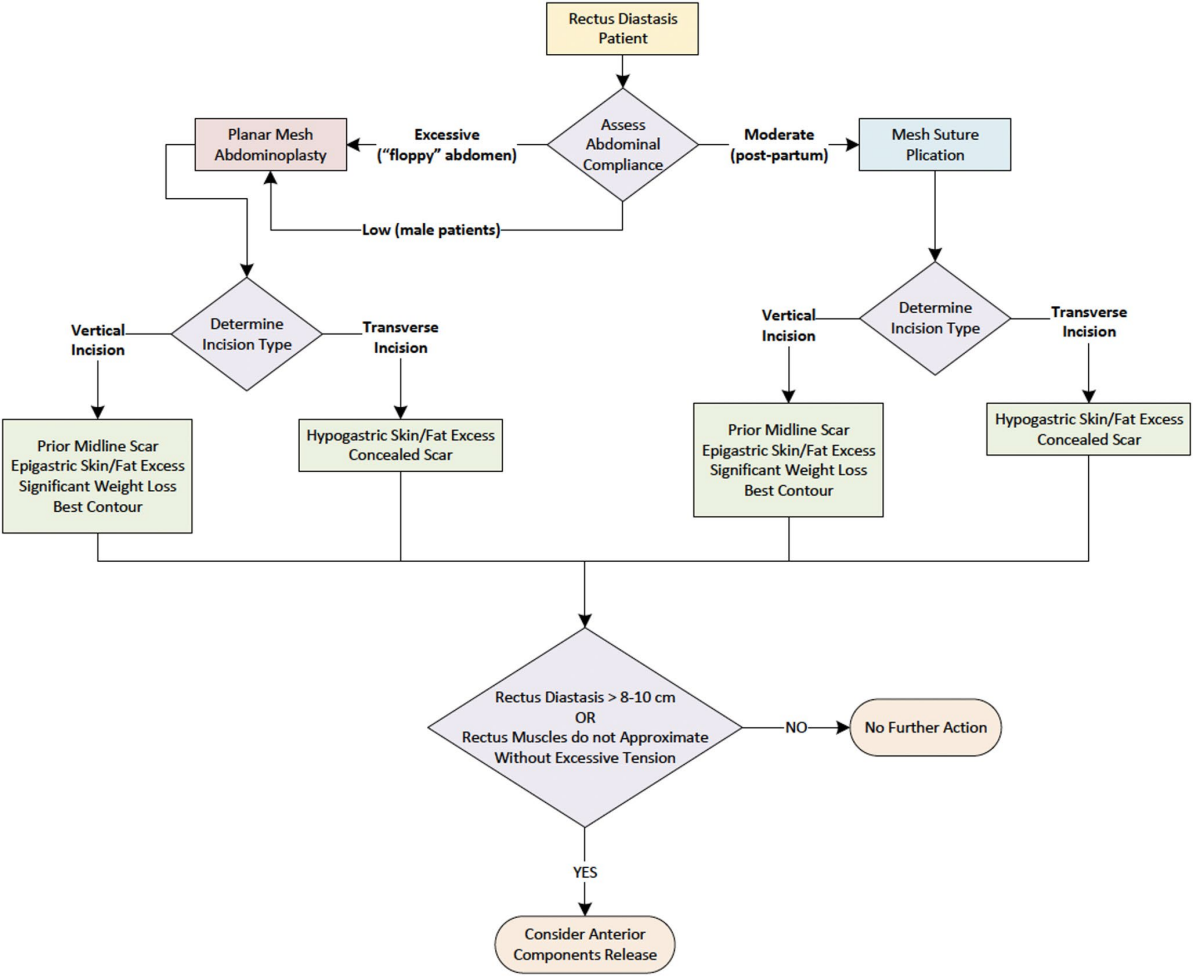
Surgical algorithm for RD with or without ventral hernia at our institution. Compliance is categorized as excessive, moderate, or low and guides the need for mesh reinforcement. Mesh suture plication is used in most moderately compliant cases; planar mesh or component separation is reserved for low compliance or wide rectus muscles. Incision type is based on scar location, tissue distribution, and contour goals. Mesh abdominoplasty = placement of well-fixed planar, polypropylene mesh in the retrorectus space along with mesh suture plication of the anterior rectus fascia

In our practice, mesh suture has become an alternative to planar mesh in patients with favorable anatomy, specifically those patients with RD but without significant rectus muscle widening. Previously, planar mesh was preferred for moderate to severe RD, but we now reserve it where rectus approximation would not appreciably narrow the semilunar lines due to excessive muscle width [33]. For others, mesh suture may represent a “sweet spot” between traditional suture and planar mesh repairs, balancing technical efficiency, tension mitigation, and aesthetic outcomes. In our practice, abdominal compliance is used as a clinical tool to guide surgical decision-making (Fig. 6). We define compliance based on the patient’s ability to engage the abdominal musculature and generate intra-abdominal pressure during physical examination, in combination with assessment of abdominal wall laxity intraoperatively. We stratify patients into three categories: excessive, moderate, and low compliance. Excessive compliance describes a soft, lax abdominal wall with minimal intrinsic tension or muscular tone, often seen in multiparous women who have had twin gestations or very large newborns. Simple approximation of the medial borders of the rectus muscle will leave a “floppy” non-aesthetic abdominal contour, as the muscles themselves have little to no tone, and thus, mesh abdominoplasty, defined as a retrorectus planar permanent mesh with mesh suture approximation of the rectus muscles, is preferred. Moderately compliant abdominal walls are pliable but with some inherent tone or resistance. In these patients, the mesh suture could meaningfully approximate the two hemi-abdomens and generate enough lasting tension to achieve an hourglass shape. Low compliance refers to a firm, non-pliable abdominal wall with high intrinsic tension, often due to well-developed rectus muscles and dense fascia. These patients may have difficulty achieving midline approximation without creating excessive tension across the closure. Male patients, especially those with athletic builds, frequently fall in this category. In this category, mesh abdominoplasty is preferred to provide durable reinforcement and optimize contour, as traditional mesh suture repair alone may not sufficiently counteract the high recoil forces of the abdominal wall. This compliance-based framework helps tailor repair strategy to the individual patient’s anatomy and physiology.

While this three-point classification of abdominal wall compliance has proven useful in guiding intraoperative decision-making and optimizing aesthetic outcomes, its clinical application remains largely qualitative and subject to surgeon interpretation. To better standardize this approach, future studies should incorporate CT imaging-based morphological assessment to evaluate parameters including rectus muscle thickness, width of diastasis, and lateral abdominal wall quality. Objective correlation between imaging findings and surgeon compliance assessments would allow for greater reproducibility and potentially enhance preoperative planning. Validation of this framework through prospective morphometric studies would further strengthen its clinical utility and support its integration into procedural algorithms.

### Complication profile and patient selection

In our study, there was a single hernia recurrence, identified 165 days postoperatively in a patient with a prior biologic mesh hernia repair and recurrent defect measuring 5 cm in transverse width, substantially larger than the cohort mean of 2.8 cm and exceeding the typical indication for mesh suture repair in this context. Upon review, we recognize that the size of this patient’s defect falls outside of the clinical scope of this series and while inclusion of this case reflected real-world surgical decision-making at the time, it introduces heterogeneity. Current principles of abdominal wall reconstruction support the use of planar mesh for larger or more complex defects, particularly those involving prior failed repairs. Before mesh suture can be considered in such contexts, its safety, efficacy, and durability must first be validated in smaller, lower risk defects. The presence of this outlier reinforces the need for clear anatomic and clinical criteria to guide patient selection and highlights the importance of future prospectively studies that stratify outcomes by defect size and complexity to optimize device selection.

Importantly, this series reflects an aesthetic surgical population undergoing elective, self-pay, abdominoplasty, rather than reconstructive procedures such as panniculectomy, which are typically performed in higher BMI patients or those with massive weight loss [34]. These differences influence surgical planning, particularly with respect to the extent of dissection, use of liposuction, application of rectus diastasis plication and expectations for contouring and these differences must be considered when interpreting outcomes. As such, findings from this study may not be generalizable to higher-risk populations, further underscoring the need for careful patient selection and context-specific interpretation of results.

Beyond patient selection, the safety and tolerability of nonabsorbable materials are also critical considerations in assessing feasibility. Concerns have been raised in the literature regarding the potential for chronic pain or foreign body sensation due to the chronic inflammatory response elicited by permanent materials [35]. In this study, no patients reported chronic pain or palpability related to mesh suture, as documented in the EMR. This favorable profile may reflect both the surgical technique (all knots were buried within the plication) and the device’s structural characteristics; under tension, mesh suture flattens to a ribbon-like configuration that conforms closely to tissue. Lastly, the mean operative time was approximately three hours, comparable to the senior author’s typical duration for abdominoplasty without mesh suture. Given that this time includes many concurrent procedures, the use of mesh suture did not appear to prolong operative time meaningfully and may offer a technically efficient solution in appropriate cases.

### Limitations

This was a single-center, retrospective study and therefore limited by its design. It is important to note that the mean BMI of this cohort was 26.6, reflective of a generally health population seeking aesthetic improvement. As such, findings may not be generalizable to higher-risk or higher-BMI populations commonly seen in the ventral hernia literature. All surgeries were performed by a single surgeon experienced with mesh suture; while this supports technical consistency, it may limit generalizability and introduces the possibility of expert bias. We acknowledge that a learning curve may exist for new users of mesh suture, though its handling is similar to standard suture. While 90-day SSI, SSE, readmission and reoperation rates were well-captured, the mean follow-up duration of less than one year is insufficient to fully assess long-term outcomes such as hernia recurrence or delayed mesh-related complications such as chronic infection. Notably recurrence often manifests beyond 18 months, and additional follow-up is needed to assess this outcome. Although not designed as a comparative study, this early experience provides feasibility and data to guide future prospective, ideally randomized trials comparing mesh suture to planar mesh or other standard techniques. Finally, we also recognize that some patients may seek care outside of the treating institution. To mitigate this, we utilized “Care Everywhere,” which provides visibility into external encounters for participating health systems; however, the scope is not universal. Longer-term follow up and larger studies will be required to more definitively evaluate the durability and broader application of mesh suture use in this study.

## Conclusion

Mesh suture appears to be a feasible technique for RD plication in the setting of abdominoplasty, with or without concurrent hernia repair or other abdominal procedures. In this small, single-surgeon series, mesh suture repair was associated with low short-term complication rates. However, given the heterogeneity of the cohort and limited follow up, particularly in cases involving hernias > 1 cm, these findings must be considered preliminary and hypothesis-generating. As an initial feasibility study, this work provides a foundation for future larger, well-controlled investigations with prospective data collection, stratification by hernia size, and longer follow-up to assess durability, recurrence risk, and broader application compared to standard repair techniques.
